# Two-day Hospital Stay After Laparoscopic Colorectal Surgery under an Enhanced Recovery after Surgery (ERAS) Pathway

**DOI:** 10.1007/s00268-013-2155-x

**Published:** 2013-07-24

**Authors:** Gustavo Rossi, Hernán Vaccarezza, Carlos A. Vaccaro, Ricardo E. Mentz, Victor Im, Adrián Alvarez, Guillermo Ojea Quintana

**Affiliations:** 1 Section of Colorectal Surgery, Service of General Surgery, Hospital Italiano de Buenos Aires, 450 Gascón st, 1181 Buenos Aires, Argentina; 2 Department of Anesthesia, Hospital Italiano de Buenos Aires, Buenos Aires, Argentina

## Abstract

**Background:**

The present study aims to examine the feasibility and safety of a two-day hospital stay after laparoscopic colorectal resection (LCR) under an enhanced recovery after surgery (ERAS) pathway.

**Methods:**

Between 2003 and 2010, 882 consecutive patients undergoing LCR were analyzed. Patients were grouped and analyzed according to whether their hospital stay was 2 days (group A) or longer (group B). Demographic, surgical, and postoperative data were compared. To identify independent predictive factors related to a short hospital stay, a multivariate analysis was also performed.

**Results:**

Group A represented 10.3 % of this series (91 patients). There were no differences regarding age, gender, BMI, ASA, and previous abdominal surgeries between groups. Group A had a lower incidence of rectal cancer and anterior resections than group B (6.6 vs. 17.7 % [*p* = 0.006] and 14.3 vs. 23.4 % [*p* = 0.048]), respectively, and a lower mean operative time (170 min vs. 192 min; *p* = 0.002). Group A had a lower overall morbidity rate than group B (5.5 vs. 16.9 %; *p* = 0.004) and a lower incidence of surgery-related complications (5.5 vs. 14.9 %; *p* = 0.001). The overall conversion rate was 10 % (only one patient in group A required conversion), and the difference in conversion rate between groups was statistically significant (1.2 vs. 10.7 %; *p* = 0.003). Group A had a lower readmission rate (0 vs. 4.9 %; *p* = 0.089). Multivariate analysis showed that conversion, postoperative morbidity, and rectal prolapse were independently associated with the length of hospital stay.

**Conclusions:**

A two-day hospital stay after LCR is safe and feasible under an ERAS pathway, without compromising the readmission or complication rate.

## Introduction

Laparoscopic colorectal surgery is becoming the standard treatment for elective colorectal resection, increasing from 13.8 % in 2007 to 42.6 % in 2009 of all colorectal resections [1]. Although laparoscopic colorectal resection (LCR) has been associated with a short hospital stay and low morbidity, mean hospital stay rates reported vary from 4 to 15 days [2–4]. A shorter hospital stay following colorectal surgery has been recently achieved through enhanced recovery after surgery programs (ERAS) [5, 6]. With this enhanced recovery protocol, some authors have mentioned a mean hospital stay between 3.5 and 4.5 days [7–9] with a low morbidity rate. However, readmission rates reported are still high, reaching up to 8–30 % [10–12]. In recent years, there has been a renewed interest in evaluating fast-track (FT) laparoscopic colorectal surgery intending to shorten hospital stay with low morbidity and readmission rates. Therefore, the aim of the present study was to examine the feasibility and safety of a two-day hospital stay after LCR in a referral center from Argentina under an ERAS protocol. A secondary outcome was to determine variables associated with a prolonged hospital stay.

## Methods

### Patients and data collection

A prospectively maintained, practice-specific database was used to identify all patients who underwent laparoscopic colorectal surgery from January 2003 to December 2010 at the Hospital Italiano de Buenos Aires, Argentina. Outcomes were prospectively recorded in an institutional review board-approved database. Patients were grouped and analyzed according to whether their hospital stay was 2 days (group A) or longer (group B). Analyzed variables included age, gender, body mass index (BMI), American Society of Anesthesiologists (ASA) score, previous abdominal surgeries, preoperative diagnosis, operation performed, postoperative outcomes, and readmission and reoperation rates.

Conversion was defined according to Chang et al. [13] as: (1) the need to perform a conventional laparotomy in order to accomplish the procedure or (2) premature abdominal incision for colorectal dissection or vascular control. All patients were analyzed based on intention-to-treat, and converted patients were included.

Postoperative morbidity was stratified according to the Dindo–Clavien classification of complications [14]. Minor surgical morbidity was considered as grades 1, 2, and 3a, and major morbidity as grades 3b, 4, and 5. Postoperative complications were divided into surgical complications (i.e., wound infection, anastomotic leak, postoperative bleeding) and general complications (i.e., cardiovascular, deep venous thrombosis). Postoperative ileus was defined according to Chen et al. [15]: if two or more episodes of vomiting of more than 200 ml occurred in the absence of a bowel movement. Resolution of postoperative ileus was defined as passage of a bowel movement in the absence of abdominal distension, nausea, or emesis.

### Surgical procedures

Preoperative mechanical bowel preparation was performed with Phosphoral for all patients. A single preoperative dose of antibiotics (oral ciprofloxacin 750 mg and intravenous ornidazole 1 g) was given. Intraoperative mechanical thromboprophylaxis was performed with intermittent pneumatic compression. Orogastric tubes were used intraoperatively and removed after surgery. Intra-abdominal drains were routinely used.

In right-sided tumors, a right colectomy was performed with the use of three ports placed at the umbilicus, the right upper quadrant, and the left iliac fossa. An alternative extra port was placed in the right iliac fossa depending on surgeon preference. Ileocolic vessels were ligated with the use of Hem-o-lok ligating clips, and the specimen was removed through a vertical midline incision above the umbilicus. An ileocolic anastomosis was performed with a continuous polypropylene 4/0 handsewn suture.

Four ports were used in left colectomy and anterior resection (umbilical, left upper quadrant, right iliac fossa, and left iliac fossa). Inferior mesenteric vessels were ligated with Hem-o-lok clips and the specimen was removed through a curved incision in the left iliac fossa or through a Pfannestiel incision, depending on surgeon preference. A colorectal anastomosis was performed with a double-stapling technique. A diverting stoma was routinely used in colorectal anastomosis located 6 cm or less from the anal verge.

### Perioperative care protocol

An enhanced recovery program was used in all cases (Table 1), including preoperative bupivacaine spinal anesthesia, early oral feeding, active mobilization, and discharge on the second postoperative day under a standardized discharge criterion. Thromboprophylaxis was performed with enoxaparin (Clexane 40 mg s.c.) starting 12 h before operation and continued once a day until hospital discharge. Oral intake and mobilization were done under a standardized program, aiming at the normal intake of fluid and solid food on the first and second postoperative days, respectively. Urinary catheters were generally removed and mobilization was started the first morning after surgery. Postoperative analgesia was provided by intravenous ketorolac. Oral analgesia was started once the oral diet was tolerated. Dietary tolerance was defined as the tolerance of two meals without nausea or vomiting. Discharge criteria included the tolerance of fluids and soft diet, adequate oral analgesia, passage of flatus or stool, and patient’s willingness to leave the hospital with adequate home support. First and second outpatient visits were planned for postoperative days 7 and 21, respectively.

**Table 1:** Enhanced recovery program

Perioperative care and discharge criteria
Preoperative care
Preadmission information and counselling
Preoperative bowel preparation
Preoperative fasting: 2 h for liquids and 6 h for solids
Preanesthesia medication
From midnight prior to surgery, patients did not receive medications known to cause long-term sedation. Patients chronically taking benzodiazepines were allowed to continue until the night prior to surgery
Short-acting medications given to facilitate insertion of the epidural catheter were accepted
Prophylaxis against thromboembolism
Subcutaneous enoxaparin 40 mg were given 12 h before the expected time of thoracic epidural catheter insertion. It was continued at 40 mg daily until discharge.
Antimicrobial prophylaxis
Patients received single-dose antibiotic prophylaxis against both anaerobes and aerobes about 1 h before surgery.
Perioperative management
Standard anesthesia protocol
Long-acting intravenous/epidural opioids were avoided in all patients unless epidural anesthesia was contraindicated.
A load dose of intravenous ketorolac (1 mg per kg body weight, calculated according to Ideal Body Weight) and a load dose of dipyrone sodium (20 mg per kg, calculated according to Ideal Body Weight) were given if not contraindicated to provide a multimodal analgesic regimen.
A midthoracic epidural commenced preoperatively containing a local anesthetic (lidocaine 2 % without epinephrine) was used unless contraindicated. Intraoperative epidural low dose fentanyl (0.5–1 μg per kg of body weight, calculated according to Ideal Body Weight) and clonidine (0.5–1 μg per kg, calculated according to Ideal Body Weight) were added to provide postoperative analgesia.
Preventing and treating postoperative nausea and vomiting
Intravenous dexamethasone 8 mg (g dose) and ondansetron 8 m (single dose) given after induction of anesthesia
Metoclopramide hydrochloride or droperidol was given if nausea or vomiting actually occurred.
Nasogastric intubation
Preventing intraoperative hypothermia
Intraoperative maintenance of normothermia with an upper-body forced-air heating cover was used routinely.
Perioperative fluid management and hemodynamic management
Preload of 500 mL of colloid was given routinely before epidural administration of local anesthetics.
Intraoperatively, lactated Ringer’s solution, 4 ml/kg per hour according to ideal body weight.
Blood loss was replaced 1:1 with colloids .
Transfusion (red cells) was given according to a preoperative target hematocrit that was defined according to age (older or younger than 65 years of age) and the presence or absence of cardiopathy. If neither of these determinants were present (cardiopathy or age older than 65) target hematocrit was 26. If only one of these factors was present, the target hematocrit was 28. Finally, if both factors were present (age older than 65 and presence of cardiopathy) the target hematocrit was 30.
Urinary drainage
Urrinay catheterization was maintained routinely for 24 h after operation
Prevention of postoperative ileus
Midthoracic epidural analgesia and avoidance of fluid overload were used to prevent postoperative ileus.
Posptoerative care
Postoperative analgesia
During the time patients stayed in the Post Anesthesia Care Unit (PACU) they received a continuous epidural midthoracic low-dose local anesthetic (0.125 % bupivacaine) and a low-dose opioid (2 mg per mL of the analgesic solution). Epidural catheters were removed before discharge from PACU.
Ketorolac 1 mg/kg (calculated according to Ideal Body Weight) was given every 8 h throughout the postoperative course.
Oral analgesia was provided when the patient was able to tolerate oral intake.
Postoperative nutritional care
Liquid diet postoperative day (POD) 1
Soft diet POD 2
Early mobilization
Patients were nursed in an environment that encouraged independence and mobilization.
Patients were strongly encouraged to be out of bed longer than 2 h beginning on the day after operation
Discharge criteria
Passing flatus or stool
Afebrile, and without tachycardia
Tolerance of oral feeding
Adequate control of pain with oral analgesia
Patient ambulating independently
Adequate support at home

### Statistical analysis

Group data of continuous variables were expressed by the mean ± standard deviation (SD). We used Student’s *t* test, the Mann–Whitney *U* test, or analysis of variance (ANOVA) for comparing means, when appropriate. Categorical variables were compared with the *χ* ^2^ test. Multivariate analysis by both logistic and multiple regression was used to identify independent variables associated with length of hospital stay, adjusting for possible confounders. Odds ratios with associated 95 % confidence intervals (CI) were calculated. Multivariate models included variables statistically associated with those in univariate analysis, as well as those considered to have clinical relevance in the primary outcome.

All statistics were two-tailed and a *p* value <0.05 was deemed significant. Statistical analysis was done with the software package NCSS 2007, PASS 2005, GESS 2066 (Hintze J, 2077, Kaysville, UT).

## Results

There were 882 patients in the analyzed period. Mean age was 63.7 years old (±14.9), females accounted for 46.9 % of the patients, and the mean BMI was 26 kg/m^2^ (±4.4). Group A represented 10.3 % of the patients in this series (91 patients). Additional demographic characteristics are shown in Table 2. There were no differences regarding age, gender, BMI, ASA, and previous abdominal surgeries between groups. The most common diagnosis of the overall population was colon cancer/polyp (55.8 %), followed by rectal cancer (16.5 %) and diverticular disease (13 %). The intraoperative data are shown in Table 3. The most frequent operations performed were sigmoidectomy (*n* = 135, 15 %), left colectomy (*n* = 133, 15 %), right colectomy (*n* = 128, 14.5 %), high anterior resection (*n* = 74, 8.4 %), low anterior resection (*n* = 48, 5.4 %), ultra-low anterior resection (*n* = 76, 8.6 %), and abdominoperineal resection (APR) (*n* = 11, 1.2 %). Group A had a lower incidence of rectal cancer and anterior resections than group B (6.6 vs. 17.7 % [*p* = 0.006] and 14.3 vs. 23.4 % [*p* = 0.048], respectively), and a lower mean operative time (170 vs. 192 min; *p* = 0.002). Table 4 shows postoperative outcomes. The median hospital stay was 3 days and there were no postoperative deaths in this series. Regarding postoperative morbidity, the overall morbidity rate was 15.6 % (139 patients), but 68 % of affected patients (*n* = 94) had minor complications (Dindo–Clavien classification 1, 2, or 3a). There were 150 postoperative complications with 123 (13.9 %) surgical complications and 27 (3 %) general complications among 882 patients. The most common surgical complication was postoperative ileus (49 patients) followed by wound infection (20 patients). Compared to group B, group A had a lower overall morbidity rate (5.5 vs. 16.9 %, respectively; *p* = 0.004) and a lower incidence of surgery-related complications (5.5 vs. 14.9 %, respectively; *p* = 0.001). Postoperative ileus was more frequent in group B than in group A (6 vs. 0 %, respectively; *p* = 0.02). None of the patients (0 %) in group A had general complications, whereas 27 patients (3.4 %) in group B had them (*p* = 0.07). Univariate analyses showed that male gender, preoperative comorbidities, and ASA score III–IV were associated with higher incidence of postoperative complications.

**Table 2:** Demographic data of 882 patients undergoing LCR

	All	2 days	3+ days	*p* value
Number of patients	882	91	791	–
Male gender, % (*n*)	52.6 % (464)	48.4 % (44)	53.1 % (420)	0.39
Mean age (SD)	63.7 (±14.9)	63.2 (±14.6)	63.7 (±14.9)	0.38
Mean BMI (SD)	26 (±4.4)	26.2 (±3.9)	25.9 (±4.5)	0.35
BMI > 30, % (*n*)	55 (483)	61.5 (56)	54 (427)	0.17
ASA III–IV, % (*n*)	27.8 (245)	24 (22)	28 (223)	0.41
Comorbidities, % (*n*)	52 (457)	48.4 (44)	52.2 (413)	0.48
Previous surgeries, % (*n*)	49 (430)	52.7 (48)	48.3 (382)	0.42
Colon cancer/polyp, % (*n*)	55.8 (493)	57.1 (52)	55.7 (441)	0.80
Rectal cancer/polyp, % (*n*)	16.5 (146)	6.6 (6)	17.7 (140)	0.006
Diverticular disease, % (*n*)	13 (113)	13.2 (12)	12.8 (101)	0.91
IBD, % (*n*)	3.7 (33)	6.1 (2)	3.9 (31)	0.41
Hartmann reversal, % (*n*)	3.6 (32)	3.3 (3)	3.7 (29)	0.85
FAP, % (*n*)	1.6 (14)	1.1 (1)	1.6 (13)	0.69
Rectal prolapsed, % (*n*)	1.4 (12)	5.5 (5)	0.9 (7)	< 0.001
Endometriosis, % (*n*)	0.9 (8)	3.3 (3)	0.6 (5)	0.01
Colonic inertia, % (*n*)	0.9 (8)	25 (2)	75 (6)	0.17
CPAC, % (*n*)	0.9 (8)	3.3 (3)	0.6 (5)	0.01
Anal cancer, % (*n*)	0.5 (4)	1.1 (1)	0.4 (3)	0.33
Colonic volvulus, % (*n*)	0.3 (3)	0 (0)	0.4 (3)	0.56
Peutz Jeghers, % (*n*)	0.3 (3)	0 (0)	0.4 (3)	0.56
Ovarian cancer, % (*n*)	0.2 (2)	0 (0)	0.3 (2)	0.63
Lymphoma, % (*n*)	0.1 (1)	0 (0)	0.1 (1)	0.73
Presacral tumor, % (*n*)	0.1 (1)	0 (0)	0.1 (1)	0.73
Ischemic colitis, % (*n*)	0.1 (1)	0 (0)	0.1 (1)	0.73

*LCR* *BMI* body mass index, *ASA* American Society of Anesthesiologists score, *SD* standard deviation, *IBP* inflammatory bowel disease, *FAP* familial adenomatous polyposis, *CPAC* colonic perforation after colonoscopy

**Table 3:** Intraoperative data

	All	2 days	3+ days	*p* value
Number of patients	882	91	791	
Mean operative time, min (SD)	190 (69)	170 (61)	192 (69)	0.001
Conversion rate, % (*n*)	10 (86)	1.2 (1)	10.7 (85)	0.003
Right colectomy, % (*n*)	25.8 (228)	32 (29)	25.2 (199)	0.14
Left colectomy, % (*n*)	15 (133)	14.4 (13)	15.2 (120)	0.85
Sigmoidectomy, % (*n*)	15 (135)	16.7 (15)	15.2 (120)	0.70
Anterior resection, % (*n*)	22.5 (198)	14.3 (13)	23.4 (185)	0.048
APR, % (*n*)	1.2 (11)	0 (0)	1.4 (11)	0.25

*APR* abdominoperineal resection

**Table 4:** Postoperative outcomes

	All	2 days	3+ days	*p* value
Median hospital stay (95 % CI)	3 (3–4)	2 (2–2)	3 (3–4)	<0.001
Postoperative mortality, % (*n*)	0	0	0	–
Morbidity, % (*n* complicated patients)	15.6 (139)	5.5 (5)	16.9 (134)	0.004
Complication grade				0.09
(Dindo–Clavien classification)				
1	33	4	29	
2	53	1	52	
3a	3	0	3	
3b	31	0	31	
4a	15	0	15	
4b	4	0	4	
Surgical complications, % (*n*)	13.9 (123)	5.5 (5)	14.9 (118)	0.001
General complications, % (*n*)	3 (27)	0 (0)	3.4 (27)	0.07
Reoperation, % (*n*)	4.2 (37)	0 (0)	4.7 (37)	0.03
Readmission, % (*n*)	4.4 (39)	0 (0)	4.9 (39)	0.08

*CI* confidence interval

The overall conversion rate in this series was 10 % (86 patients). Only one patient in group A required conversion; thus when compared with group B, group A had a lower incidence of conversion (1.2 vs. 10.7 %; *p* = 0.003). The overall readmission rate was 4.4 % (39 patients); there were no readmissions in group A. Although readmission rate in group A was lower than in group B (0 vs. 4.9 %) this difference had no statistical significance (*p* = 0.089). Another finding of the univariate analyses was that patients with BMI >30 and low anterior resection had a higher readmission rate (8.5 vs. 3.7 % [*p* = 0.049] and 12.5 vs. 4 % [*p* = 0.019], respectively). Thirty-seven patients underwent reoperation. None of the patients in group A required reoperation, whereas the reoperation rate in group B was 4.7 % (*p* = 0.035). Indications for reoperation were peritonitis in 12 patients (32 %), intestinal occlusion in 11 patients (29 %), postoperative bleeding in 6 patients (16 %), anastomotic leak in 4 patients (10 %), intra-abdominal abscess in 2 patients (5 %), acute laparotomy dehiscence in 1 patient (2 %), and intestinal ischemia 1 patient (2 %).

Multivariate analysis showed that conversion, postoperative morbidity, and rectal prolapse were independently associated with a 2-day hospital stay, after adjusting for age, gender, BMI <30, ASA III-IV, rectal polyp/cancer, anterior resection, operative time, and colonic perforation after colonoscopy (CPAC) (Table 5).

**Table 5:** Multivariate analysis of variables related to length of hospital stay

	Odds ratio	95 % CI	*p* value
Female gender	0.93	0.58–1.47	0.76
Age	1	0.98–1.02	0.84
BMI > 30	1.07	0.56–2.06	0.83
ASA III–IV	1.16	0.67–2.05	0.60
Rectal polyp/cancer	2.68	0.9–7.97	0.77
Anterior resection	0.86	0.38–1.91	0.71
Operative time	1	0.99–1	0.32
Conversion	7.37	0.99–54.8	0.05
Postoperative morbidity	3	1.17–7.75	0.02
Rectal prolapsed	0.17	0.52–0.58	0.005
CPAC	0.29	0.06–129	0.10

## Discussion

Despite the fact that LCRs allow an earlier recovery and discharge from hospital, a short hospital stay has not been routinely achieved [10]. Moreover, some authors have reported short hospital stays after open colorectal surgery when combining fast-track or multimodal recovery programs. In this regard, Behrns et al. [16] and Delaney et al. [17] reported a mean hospital stay of 4.4 and 3.5 days, respectively, after open surgery. Basse et al. [7] pushed these results further and reported 2-day hospital stays in a randomized trial comparing open versus laparoscopic surgery, showing no differences between groups. However, morbidity and readmission rates reported reached up to 27 and 12 %, respectively. In accordance with these data, Andersen et al. [18] mentioned a decrease in the readmission rate from 20.1 to 11.3 % when comparing patients with a 2-day versus a 3-day planned hospital stay. On the other hand, the LAFA study compared postoperative outcomes in four groups (2 laparoscopic and 2 open colectomy groups with and without an FT program). The median hospital stay of patients undergoing laparoscopic surgery with FT care was shorter than the other groups (laparoscopic/FT: 5 days, open/FT: 7 days, laparoscopic/standard: 6 days, and open/standard: 7 days (*p* < 0.001). However, there were no differences among groups regarding postoperative morbidity and mortality, reoperation and readmission rates, and quality of life at 2 and 4 weeks. The authors concluded that the optimal operative treatment for patients requiring segmental colectomy for colon tumor resection is laparoscopic embedded in a FT program or accelerated recovery [19]. A recent report from Delaney et al. [10] mentioned an overall readmission rate of 8.5 %, with 5.4 and 7.7 % after a 2 and 3-day hospital stay, respectively, in a series of 118 patients treated laparoscopically. However, this represents a single-institution series and additional data are needed. In the same way, Levy et al. [20] reported a series of 10 patients who underwent LCR with a 23 h hospital stay and no readmissions.

The results presented in this study bring new evidence supporting feasibility and safety of short hospital stays following LCR. Our series of 882 patients shows a median hospital stay of 3 days, with 10 % of patients discharged within the first 48 h. Readmission rates were 0 % for patients discharged on the second postoperative day and 4.9 % for patients who stayed longer than 2 days. Moreover, patients discharged within 48 h after surgery had lower morbidity and reoperation rates. Of course early discharge does not determine a lower morbidity, reoperation, and readmission rates, and it is likely these findings are related to several variables, including patient factors and disease factors, surgical experience, and procedure-specific issues. However, it allows patients who are recovering well from surgery on the second postoperative day to have a better chance of not having postoperative complications. Thus, colorectal surgeons should try to identify these patients in order to provide optimal postoperative care with appropriate efficiency. Therefore, FT recovery programs and standardized discharge criteria are of the utmost importance.

In our series, patients discharged on the second postoperative day had a lower mean operative time, a lower incidence of rectal cancer, and hence, a lower incidence of anterior resection. There were no differences between groups A and B regarding high and ultra-low anterior resections (8.8 vs. 8.3 % [*p* = 0.9] and 4.4 vs. 9.1 % [*p* = 0.12], respectively). However, compared to group B, group A had a lower proportion of low anterior resections (5.9 vs. 1.1 % [*p* = 0.05], respectively), which were associated with a higher readmission rate in a subanalysis of these data. This higher readmission rate associated with low anterior resection could reflect potentially severe complications (e.g., anastomotic leaks), which usually have an asymptomatic course in patients with a diverting stoma, which is routinely performed in an ultra-low anterior resection. It is also worth mentioning that while group A had a higher proportion of rectal prolapse, CPAC, and endometriosis compared to group B, the small sample size makes it impossible to draw any conclusions from these numbers.

Multivariate analysis showed that after adjusting for confounding factors, conversion, postoperative morbidity, and rectal prolapse were independently associated with length of hospital stay. An important issue to consider when analyzing postoperative outcomes of LCR, is the impact of conversion on postoperative results. It is known that conversion is associated with prolonged operative time, increased morbidity, slower recovery, and prolonged hospital stay [21–23]. Senagore et al. [12], in a series of 181 laparoscopic sigmoidectomies with a conversion rate of 12.1 %, reported a mean hospital stay of 2.9 ± 1.2 days for laparoscopically completed cases versus 6.4 ± 1.4 days for converted cases. They did not, however, report the overall mean hospital stay. Overall, patients with complications often required additional pharmacological treatments or surgical procedures that determine a longer hospital stay. Regarding this issue, postoperative ileus has been identified as the most frequent surgical complication associated with delayed hospital discharge, and laparoscopy has been claimed to reduce postoperative ileus [15]. Interestingly, patients in group A had a lower incidence of postoperative ileus than patients in group B, and there was no association between postoperative ileus and the operation performed. Similar results were reported by Delaney et al. [10]. The small number of rectal prolapse cases makes it difficult to establish why rectal prolapse is associated with earlier hospital discharge. However, we can infer that this is determined by technical aspects of the operation, such as less dissection of the descending colon or the avoidance of unnecessary resections.

Finally, one important argument against fast-track recovery programs is associated with the necessity of home care nursing [24]. However, our study shows that no skilled nursing was required after early discharge following LCR when applying standardized perioperative care programs. Moreover, ERAS programs are often criticized for difficulty in assessing patient compliance [25], and this could be cited as a limitation of our study. Future institutional efforts will be required to oversee this critical aspect of perioperative care.

In conclusion, a two-day hospital stay after LCR is safe and feasible under an ERAS pathway. Patients fulfilling standardized criteria can be safely discharged on the second postoperative day with a low readmission and complication rate. Both conversion to open conventional surgery and postoperative morbidity, however, were associated with prolonged hospital stay. 
